# Frequency synchronization induced by frequency detuning

**DOI:** 10.1126/sciadv.adu4114

**Published:** 2025-06-11

**Authors:** Jorge Luis Ocampo-Espindola, Christian Bick, Adilson E. Motter, István Z. Kiss

**Affiliations:** ^1^ Department of Chemistry, Saint Louis University, St. Louis, MO 63103, USA.; ^2^ Center for Network Dynamics, Northwestern University, Evanston, IL 60208, USA.; ^3^ Department of Physics and Astronomy, Northwestern University, Evanston, IL 60208, USA.; ^4^ Department of Mathematics, Vrije Universiteit Amsterdam, Amsterdam 1081 HV, Netherlands.; ^5^ Mathematical Institute, University of Oxford, Oxford OX2 6GG, UK.; ^6^ Department of Mathematics, University of Exeter, Exeter EX4 4QF, UK.; ^7^ Institute for Advanced Study, Technische Universität München, Garching 85748, Germany.; ^8^ Department of Engineering Sciences and Applied Mathematics, Northwestern University, Evanston, IL 60208, USA.; ^9^ Northwestern Institute on Complex Systems, Northwestern University, Evanston, IL 60208, USA.

## Abstract

It is widely held that identical systems tend to behave similarly under comparable conditions. Yet, for systems that interact through a network, symmetry breaking can lead to scenarios in which this expectation does not hold. Prominent examples are chimera states in multistable phase-oscillator networks. Here, we show that for a broad class of such networks, asynchronous states can be converted into frequency-synchronized states when identical oscillators are detuned to have different intrinsic frequencies. We show that frequency synchronization is achieved over a range of intrinsic frequency detuning and is thus a robust effect. These results, which are supported by theory, simulations, and electrochemical oscillator experiments, reveal a counterintuitive opportunity to use parameter heterogeneity to promote synchronization.

## Teaser

Analyses and experiments on multistable networks show that frequency detuning can promote synchronization.

## INTRODUCTION

Synchronization of coupled oscillators is an emergent network phenomenon, which enjoys remarkable properties and has far-reaching implications in natural and engineered systems (*1*, *2*). Among the factors governing this phenomenon, coupling takes center stage as it can stabilize otherwise unstable synchronization states (*3*). However, coupling can also lead to instabilities and impede synchronization even when the oscillators are identical and identically coupled (*4*, *5*). A fundamental problem of current interest is the interplay between the coupling network and other factors governing synchronization, which include the initial conditions and oscillator parameters (*6*–*8*). The former plays a central role in the emergence of a rich variety of spatiotemporal patterns (*6*, *9*), including many instances of cluster synchronization and chimera states (*10*, *11*). An outstanding question in this context concerns the impact of oscillator parameters on such patterns.

Intuitively, parameter heterogeneity across oscillators may be expected to inhibit synchronization. This expectation is supported by studies of simple phase oscillator models, where frequency detuning is observed to increase the coupling threshold for synchronization (*12*) or even destroy synchronization (*13*). However, recent research on higher-dimensional oscillator models shows evidence that heterogeneity can serve as a stabilizing element in synchronization (*14*, *15*). This is different from earlier work on synchronization enhancement, which was centered on the manipulation of coupling network (*16*), control of dynamical variables (*17*), and homogenization of oscillator inputs (*18*). There are now various studies showing the beneficial impact of heterogeneity in applications as diverse as parametric wave amplification (*19*), persistency of oscillations in active matter (*20*), chaos control in multilayer networks (*21*), cluster synchronization (*22*), state stabilization in optical resonators (*23*), phase-locking in laser arrays (*24*), neural computation (*25*), and even quantum dynamics (*26*). It remains an open question, however, whether frequency detuning can steer spatiotemporal patterns and promote global synchronization in phase oscillator networks. A phase oscillator description would offer promising new directions for theoretical advancements (*27*), enabling extensions to novel types of networks and oscillators. It would also open avenues for experiments where phase models have proven effective in exploring emerging spatiotemporal pattern formations (*11*, *28*, *29*).

Here, starting from states in which identically coupled identical oscillators do not synchronize, we show how frequency synchronization can be achieved robustly when the phase oscillators are detuned. Our results are established for multistable networks of weakly coupled oscillators that, upon phase reduction, exhibit second-harmonic coupling and phase frustration. Our approach leads to the synchronization of otherwise asynchronous states that coexist with synchronous ones. The results are further demonstrated for neuronal and chemical oscillators, with experimental validation conducted specifically for electrochemical systems. For concreteness, we first focus on modular networks consisting of two populations of *N* oscillators each, with all-to-all intrapopulation coupling of strength *K* > 0 and weaker all-to-all inter-population coupling of strength ε*K* ≥ 0 [as considered in numerous previous studies (*30*–*34*)]. Notably, we show that the effect can be achieved by manipulating a single independent parameter in each population that is out of synchrony with the others, making it scalable to large networks. This represents an important step toward reducing computational complexity, which could otherwise require specific parameter adjustments of individual oscillators.

**Fig. 1.**
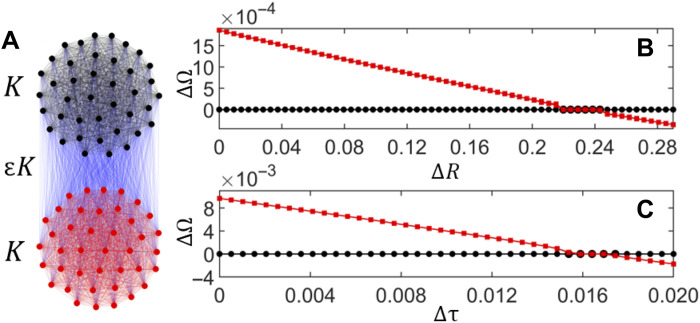
Detuning-induced frequency synchronization for two oscillator network models. (**A**) Modular network exhibiting all-to-all intra- and interpopulation coupling of strengths *K* and ε*K*, respectively. (**B** and **C**) Frequency of individual oscillators in population 1 (black) and population 2 (red), relative to oscillator 1 in population 1, as a function of the detuning parameter for: chemical oscillators with electrical coupling in (B) and the integrate-and-fire model with a refractory period in (C). The detuning is introduced in resistances (ΔR) in (B) and relaxation timescales (Δτ) in (C), in both cases for *N* = 2 oscillators in each population. For model description and other parameters, see the Supplementary Text.

The essence of the phenomenon is illustrated in Fig. 1 for two oscillator models, namely, a kinetic chemical oscillator model (*35*) and an integrate-and-fire neuronal model (*36*), for *N* = 2. In both cases, when the oscillators are identical, the system exhibits a type of cluster-synchronized state, also known as a weak chimera (*37*, *38*), in which each population is synchronized to a different frequency. However, the frequency difference is gradually reduced as a parameter effectively controlling the intrinsic frequencies is varied in one of the populations. Global frequency synchronization is then achieved when the intrinsic frequencies are suitably detuned between the two populations. This state, in which the two populations exhibit identical frequencies, is stable not just for a specific parameter assignment but rather over a range of intrinsic frequency detuning. Motivated by such simulation results, here we first establish a theory to identify the mechanisms and conditions that can give rise to this effect. We also demonstrate the robustness of the effect in modular networks with more general structures, including variations in coupling density, population number, and population sizes. We then report on experiments in electrochemical oscillator networks to test and expand on our predictions in a realistic setting.

## RESULTS

### Phase-model theory predicts detuning-induced synchronization

The theory is established using a phase oscillator model, which is a suitable mathematical description for the dynamics of a broad class of weakly coupled systems (*12*, *39*), including those in our simulations and experiments (*35*, *36*). To elucidate the dynamical mechanisms that give rise to the emergence of frequency synchronization, we consider two-population networks with N=2n oscillators in each population for arbitrary n≥1 (Fig. 1A). The state of oscillator k∈{1,…,N} in population σ∈{1,2}, or simply oscillator (σ,k), is given by a phase variable $\theta_{\sigma }^{k}$. The oscillator phases evolve according to

$${\dot{\theta }}_{\sigma }^{k}=\omega_{\sigma }+\frac{K}{N}\sum_{j=1}^{N}g\left(\theta_{\sigma }^{j}-\theta_{\sigma }^{k}\right)+\frac{\varepsilon K}{N}\sum_{j=1}^{N}g\left(\theta_{\kappa }^{j}-\theta_{\sigma }^{k}\right)$$ (1)

where κ=1+(σ mod 2), $\omega_{\sigma }$ is the intrinsic frequency of the oscillators in population σ, and *g* is a 2π-periodic function mediating the interaction between the oscillators. We focus on 0≤ε<1 and assume, without loss of generality, that *K* = 2. Consider the trajectory $\theta_{\sigma }^{k}\left(t\right)$ for an initial condition $\left[\theta_{1}^{1}\left(0\right),\ldots ,\theta_{1}^{N}\left(0\right),\theta_{2}^{1}\left(0\right),\ldots ,\theta_{2}^{N}\left(0\right)\right]=:\theta_{\left(0\right)}$. The asymptotic average angular frequency along the trajectory is $\Omega_{\sigma }^{k}\left[\theta_{\left(0\right)}\right]={\text{lim}}_{T\to \infty }\frac{1}{T}\theta_{\sigma }^{k}\left(T\right)$. Oscillators (σ,k) and (κ,j) are frequency synchronized for the trajectory with initial condition $\theta_{\left(0\right)}$ if $\Omega_{\sigma }^{k}\left[\theta_{\left(0\right)}\right]=\Omega_{\kappa }^{j}\left[\theta_{\left(0\right)}\right]$.

Since the oscillators in each population are identical and also identically coupled, there are symmetries that restrict the dynamics of Eq. 1 (*40*, *41*). These symmetries imply that all oscillators within a single population have the same average angular frequency $\Omega_{\sigma }^{k}\left[\theta_{\left(0\right)}\right]=:\Omega_{\sigma }\left[\theta_{\left(0\right)}\right]$ ∀k (*37*, *42*). This allows us to define the frequency difference between populations as ${\Delta \Omega }_{21}\left[\theta_{\left(0\right)}\right]≔\Omega_{2}\left[\theta_{\left(0\right)}\right]-\Omega_{1}\left[\theta_{\left(0\right)}\right]$. The symmetries of the system also give rise to invariant subsets in which the oscillators in each population are partitioned into clusters of identical phases. In particular, a state in which population 1 is fully phase synchronized (all oscillators have phase $\vartheta_{1}$) and population 2 is in a balanced two-cluster configuration (half of the oscillators in phase $\vartheta_{2}$ and the other half in phase $\vartheta_{2}^{\prime }$) will retain this cluster configuration as phases evolve according to Eq. 1. This means that the set of such states

$$S≔\left\{\theta_{1}^{k}=\theta_{1}^{n+k}=\vartheta_{1},\theta_{2}^{k}=\vartheta_{2},\theta_{2}^{n+k}=\vartheta_{2}^{\prime },k=1,\ldots ,n\right\}$$

is invariant under time evolution.

In the following, we consider the cluster dynamics on S, which can be written in terms of two phase differences: $\psi ≔\vartheta_{2}-\vartheta_{1}$ and $\psi \prime ≔\vartheta_{2}^{\prime }-\vartheta_{1}$. We introduce $\Delta \omega_{12}≔\omega_{1}-\omega_{2}$ to quantify the detuning of the intrinsic frequencies between the populations. From Eq. 1, it follows that

$$\begin{array}{ll} \dot{\psi } & =-\Delta \omega_{12}-g\left(0\right)+g\left(\psi \prime -\psi \right)\\ & \;+\varepsilon \left[2g\left(-\psi \right)-g\left(\psi \right)-g\left(\psi \prime \right)\right] \end{array}$$ (2a)

$$\begin{array}{ll} \overset{˙}{\psi }\prime & =-\Delta \omega_{12}-g\left(0\right)+g\left(\psi -\psi \prime \right)\\ & \;+\varepsilon \left[2g\left(-\psi \prime \right)-g\left(\psi \right)-g\left(\psi \prime \right)\right] \end{array}$$ (2b)

which describes the cluster dynamics in the corotating reference frame of population 1. Identifying the initial conditions $\theta_{\left(0\right)}\in S$ with the corresponding initial phase differences $\psi_{\left(0\right)}≔\left[\psi \left(0\right),\psi \prime \left(0\right)\right]$, we have $\begin{array}{c} {\Delta \Omega }_{21}\left[\psi_{\left(0\right)}\right]≔{\Delta \Omega }_{21}\left[\theta_{\left(0\right)}\right]={\text{lim}}_{T\to \infty }\frac{1}{T}\psi \left(T\right) \end{array}$.

If the two populations have identical intrinsic frequencies (i.e., $\Delta \omega_{12}=0$), then, according to Eq. 2, states in which all oscillators are in-phase (i.e., ψ=ψ′=0) are stationary solutions of the dynamics. Consequently, ${\Delta \Omega }_{21}\left(0,0\right)=0$, and the populations are frequency synchronized. By contrast, if the intrinsic frequencies of the two populations are nonidentical (i.e., $\Delta \omega_{12}\neq 0$), in general, no in-phase synchronized equilibria exist. The question then is whether frequency synchronization can be achieved between two populations with nonidentical intrinsic frequencies.

To answer this question we consider the coupling function

g(ϕ)=sin(ϕ+α)−rsin(2ϕ+2α) (3)

where r>0 controls the strength of the second harmonic and α is a phase frustration parameter. Except when noted otherwise, we set $\alpha =-\frac{\pi }{2}$, which allows different cluster synchronous states, including weak chimeras, to be stable (*34*). Two results follow.

First, for frequency synchronized populations with ψ=ψ′, the frequency synchrony is maintained for weak detuning $\Delta \omega_{12}$ even if the populations are out of phase (i.e., ψ≠0); this can be related to classical studies of frequency entrainment of nonidentical oscillators (*43*). Let SS denote the points in S with ψ=ψ′. We have ${\Delta \Omega }_{21}\left(\psi ,\psi \prime \right)=0$ for (ψ,ψ′) in SS if $\left(\psi ,\psi \prime \right)=\left(\psi^{*},\psi^{*}\right)$ is an equilibrium point, which is by Eq. 2 equivalent to $\Delta \omega_{12}+2\varepsilon \left[g\left(\psi^{*}\right)-g\left(-\psi^{*}\right)\right]=0$. For our specific choice of *g*, this condition for frequency synchronization is given by $\Delta \omega_{12}=-4\varepsilon r\text{sin}\left(2\psi^{*}\right)$ and is satisfied for some $\psi^{*}$ provided that

$$|\;\Delta \omega_{12}\;|\leq 4\varepsilon r$$ (4)

**Fig. 2.**
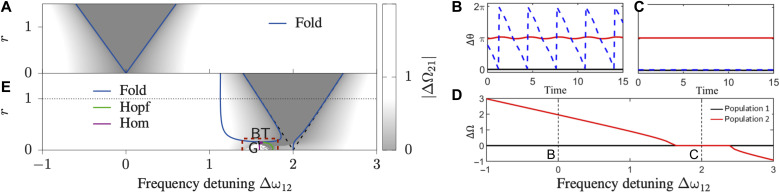
Emergence of frequency synchronization in detuned phase oscillator networks. (**A**) Bifurcation diagram for synchronization in the $\Delta \omega_{12}$ versus *r* space for the initial condition $\psi_{\left(0\right)}=\left(0.1,0.1\right)$: condition in Eq. 4 (indistinguishable from blue lines), AUTO numerical analysis (blue lines), and direct simulations (shading). (**B**) Time series of the phase differences for a weak chimera when $\Delta \omega_{12}=0$, where the curves indicate $\Delta \theta =\theta_{\sigma }^{k}-\theta_{\kappa }^{j}$ in population 1 (black line), in population 2 (red line), and between the two populations (blue dashed line). (**C**) Phase differences for a globally synchronized state induced by frequency detuning $\Delta \omega_{12}=2$ (lines as in B). (**D**) Frequency difference between populations as the detuning parameter $\Delta \omega_{12}$ is varied, where the dashed lines mark the points corresponding to (B) and (C). (**E**) Bifurcation diagram showing the emergence of synchronization induced by detuning for $\psi_{\left(0\right)}=\left(0,-\pi \right)$: condition in Eq. 5 (dashed lines), AUTO numerical analysis (solid lines), and direct simulations (shading), where the bifurcations marked are fold (blue), Hopf (green), and homoclinic (purple). For small *r*, the diagram shows a complex bifurcation structure with a region of frequency-synchronized periodic orbits, a Bogdanov-Takens bifurcation [BT; $\left(\Delta \omega_{12},r\right)\approx \left(1.588,0.160\right)$], and a global bifurcation of two homoclinic orbits [G; $\left(\Delta \omega_{12},r\right)\approx \left(1.594,0.126\right)$]. For clarity, a detailed zoom of the red dashed region is presented in fig. S5. The other parameters are ε = 0.1 in all panels, *r* = 1 in (B) to (D), and *N* = 2 in the simulations.

The condition in Eq. 4 is exact and defines an Arnold-like tongue in the $\Delta \omega_{12}$ versus *r* parameter space, as depicted in Fig. 2A. The boundaries of this region of frequency synchronization are given by fold (saddle-node) bifurcations. Therefore, this shows how detuning can maintain frequency synchronization or result in frequency desynchronization.

Second, when the two populations are not frequency synchronized with each other in S, frequency detuning can induce global frequency synchronization, as shown next. Without detuning (i.e., $\Delta \omega_{12}=0$), the system can exhibit weak chimeras, which are characterized by the lack of frequency synchronization between the two populations [i.e., ${\Delta \Omega }_{21}\left(\theta_{\left(0\right)}\right)\neq 0$] (*34*, *37*). A weak chimera is shown in Fig. 2B: population 1 is synchronized in-phase, the clusters in population 2 are synchronized with a phase difference Δψ≔ψ−ψ′≈π, and the phase differences between the populations increase monotonically. For ε=0 and $\omega_{1}=\omega_{2}=\omega$, the asymptotic frequency difference between the populations can be calculated explicitly. In this case, for $\theta_{\left(0\right)}\in S$ with Δψ=π, we have $\Omega_{1}\left[\theta_{\left(0\right)}\right]=\omega +2g\left(0\right)$ and $\Omega_{2}\left[\theta_{\left(0\right)}\right]=\omega +g\left(0\right)+g\left(\pi \right)$. This results in ${\Delta \Omega }_{21}\left[\theta_{\left(0\right)}\right]=-g\left(0\right)+g\left(\pi \right)=2≕{\Delta \Omega }_{21}^{u}>0$ for the frequency difference between the two uncoupled populations.

Now we consider the effect of intrinsic frequency detuning (i.e., $\Delta \omega_{12}>0$) for nonzero coupling between the populations (i.e., ε>0). As $\Delta \omega_{12}$ is increased for fixed initial conditions, the frequency difference ${\Delta \Omega }_{21}$ between the populations reduces and then vanishes for a range of $\Delta \omega_{12}$ values around ${\Delta \Omega }_{21}^{u}$. This is illustrated in Fig. 2 (C and D) for ε=0.1 and *r* = 1, and it reproduces closely the behavior anticipated for the models in Fig. 1.

To derive a theoretical condition for frequency synchronization induced by frequency detuning, we focus primarily on the regime ε≪1 and large *r*. In this case, there is a weak chimera close to Δψ=π in S, as described above. Let SC denote the points in S with Δψ=π. In the same spirit as in the derivation of Eq. 4, imposing the existence of an equilibrium $\left(\psi ,\psi \prime \right)=\left(\psi^{*},\psi^{*}-\pi \right)$ in SC for Eq. 2 gives an approximate condition for frequency synchronization. The condition for an equilibrium to exist is that $\Delta \omega_{12}+g\left(0\right)-g\left(\pi \right)+\varepsilon \left[g\left(\psi^{*}\right)-g\left(-\psi^{*}\right)+g\left(\psi^{*}-\pi \right)-g\left(-\psi^{*}+\pi \right)\right]=0$.

For our choice of *g* and the assumption that *r* is large, an equilibrium exists provided that

$$|\;\Delta \omega_{12}-{\Delta \Omega }_{21}^{u}\;|\leq 4\varepsilon r$$ (5)

This condition gives a good approximation for the numerically computed fold bifurcation lines in Fig. 2E. Our numerical bifurcation analysis was implemented using AUTO (*44*) and also shows that additional local and global bifurcations occur for r<1. Nevertheless, the condition in Eq. 5 provides a good approximation for r≳1.

### Extension to more general interaction functions

We have shown that the phase model in Eq. 1 with the interaction function *g* in Eq. 3 can exhibit frequency synchronization with frequency detuning for α=−π/2 (i.e., large phase frustration) and r≳1 (i.e., large second harmonic in *g*). However, the phenomenon is also expected for other α and *r* values, and more general *g* with independent phase shifts for the first and second harmonics. As shown in Materials and Methods, an interaction function with up to two Fourier harmonics in *g* is expected to produce frequency synchronization with detuning whenever the one- and two-cluster states are stable, and there is a frequency difference between these two states in the absence of detuning. For *g* in Eq. 3 in the range −π/2≤α<−π/4, we show that the one- and two-cluster states are stable for

$$r>r_{c}≔-\text{cos}\left(\alpha \right)/\left[2\text{cos(}2\alpha )\right]$$ (6)

we also show that the frequency difference between the one- and two-cluster states is ${\Delta \Omega }_{21}^{u}=-2\text{sin}\left(\alpha \right)$ and thus always nonzero for this range of α. For such conditions, the width of the Arnold tongue can be approximated as

$$|\;\Delta \omega_{12}-{\Delta \Omega }_{21}^{u}\;|\leq 4\varepsilon r\text{cos}\left(2\alpha \right)$$ (7)

which generalizes Eq. 5 as a condition for detuning-induced frequency synchronization. Therefore, the effect is expected in the entire (α,r) domain above where the one- and two-cluster states are stable, which was confirmed with numerical simulations in fig. S6.

Equation 6 also has another implication. For systems with large phase frustration, α≈−π/2, a second harmonic with a relatively small amplitude r>0 is sufficient for detuning-induced frequency synchronization. However, when the phase frustration parameter is smaller in magnitude (α→−π/4), a large second harmonic *r* is required for detuning-induced frequency synchronization ($r_{c}\to \infty$, see fig. S6).

We numerically calculate the phase interaction functions for the chemical oscillator and integrate-and-fire models in Fig. 1. The results, presented in fig. S7, are in agreement with the theoretical prediction: For both models, the calculated *g* function implies stable one- and two-cluster states with nonzero frequency differences. In these examples, *g* has a dominant first-harmonic and weak second-harmonic component, which is one of the shapes of *g* predicted theoretically to lead to detuning-induced frequency synchronization.

### Consistent behavior across more complex networks

**Fig. 3.**
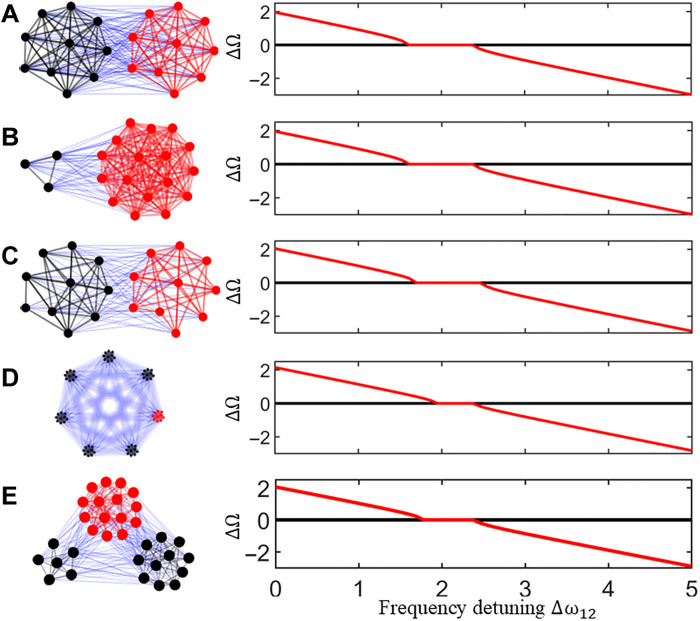
Detuning-induced frequency synchronization in increasingly complex network structures. Modular networks with varying structures (left) exhibit detuning-induced frequency synchronization (right). (**A**) Reference case: two populations with *N* = 10. (**B**) Two populations with 3 oscillators in population one and 17 in population two. (**C**) Two populations with *N* = 10 and 40% of edges randomly removed. (**D**) Seven populations with *N* = 10. (**E**) Three populations with different sizes (6, 11, and 15 oscillators) and 40% random edge removal. Black and red indicate one- and two-cluster states, respectively. Frequencies are shown relative to the first oscillator in the first one-cluster population. See the Materials and Methods for model details and parameter choices.

Thus far, the theoretical and numerical results presented are based on all-to-all modular networks composed of two strongly coupled populations that interact through weak interpopulation coupling. However, we can show that our main results extend to more general network structures, as shown in Fig. 3, where we vary the number and relative size of the populations and the coupling density. For better comparisons across the different scenarios, the coupling is assumed to occur through mean fields (i.e., the strengths of the input intra- and interpopulation couplings to a node are normalized by the corresponding degrees); for further details of the model and parameter choices, we refer to the Materials and Methods. As a reference, Fig. 3A shows that detuning-induced frequency synchronization readily occurs for an all-to-all modular network with two populations and *N* = 10, as expected from the results above.

We first examine the impact of varying the number of nodes within each population. As shown in Fig. 3B, frequency synchronization persists even with a strong population imbalance, where one population contains only 3 oscillators and the other 17 oscillators. This robustness arises from the mean-field nature of the interactions, which makes the dynamics largely insensitive to population size. Figure S8 shows additional examples across different population ratios. A similar invariance is observed when randomly removing edges in networks with equally sized populations. As illustrated in Fig. 3C, frequency synchronization remains intact even with 40% of edges removed. Figure S9 confirms this behavior for different edge removal percentages. As long as one- and two-cluster states form within each population, mean-field interactions dominate, preserving synchronization. However, when the network is further sparsified, some nodes begin to decouple from the main clusters, and detuning-induced synchronization becomes limited to connected subnetworks.

Figure 3D illustrates the effect of increasing the number of populations: A single two-cluster population is coupled to six one-cluster populations. While detuning can still induce synchronization, the width of the Arnold tongue is noticeably reduced. This trend is further supported by fig. S10, which shows that as the number of one-cluster populations increases, the Arnold tongue narrows progressively. This reduction arises because the mean field experienced by each population becomes increasingly dominated by the one-cluster dynamics. Last, the phenomenon persists even when relative population size, edge density, and number of populations are varied concurrently. This is illustrated in Fig. 3E for a heterogeneous network of three populations, with different sizes and 40% of edges randomly removed, where detuning-induced frequency synchronization still emerges, highlighting the robustness of the phenomenon.

### Experimental demonstration in electrochemical oscillators networks

**Fig. 4.**
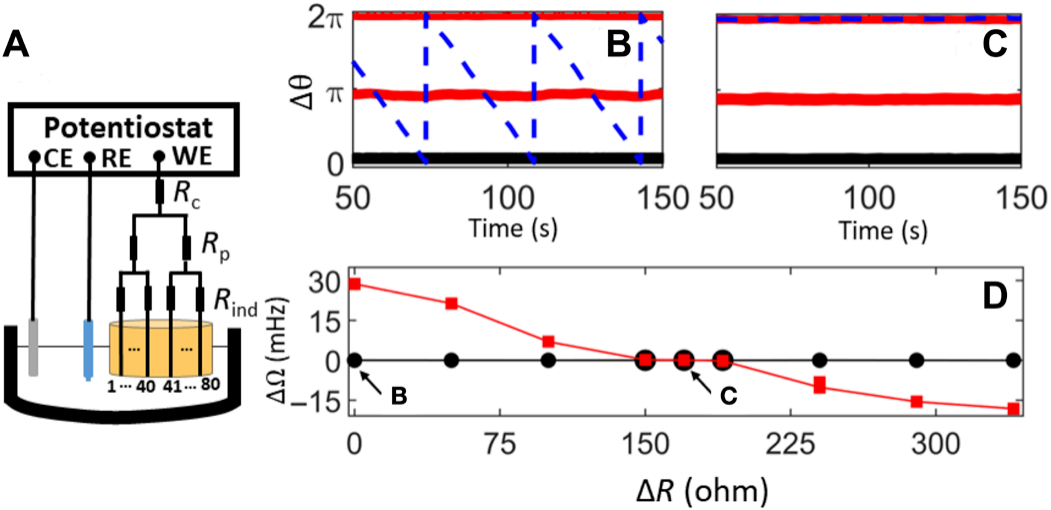
Detuning-induced frequency synchronization in electrochemical oscillator experiments. (**A**) Experimental setup for two groups of *N* = 40 oscillators, where CE, RE, and WE are the counter, reference, and working electrodes, respectively. The oscillators are coupled through a combination of individual ($R_{\text{ind}}$), population ($R_{p}$), and global ($R_{c}$) resistances. (**B**) Phase differences for a desynchronized (weak chimera) state in the absence of detuning (ΔR=0). (**C**) Phase differences for a globally synchronized state with detuning (ΔR=170 ohm). In (B) and (C), the curves indicate $\Delta \theta =\theta_{\sigma }^{k}-\theta_{\kappa }^{j}$ in population 1 (black line), in population 2 (red line), and between the two populations (blue dashed line). (**D**) Frequency of individual oscillators in population 1 (black) and population 2 (red) relative to oscillator 1 in population 1 as a function of ΔR. The states in (B) and (C) are indicated by the arrows and animated in the movie S1.

We demonstrate the emergence of frequency synchronization induced by detuning in experiments with oscillatory nickel electrodissolution in 3 M $H_{2}{\text{SO}}_{4}$ electrolyte (Fig. 4A). The nodes of our network are formed by 80 1-mm–diameter wires, each connected to a potentiostat (ACM Instruments, Gill AC). When the potentiostat applies a constant circuit potential (*V* = 1.260 V) with respect to a reference electrode ($\text{Hg}/{\text{Hg}}_{2}{\text{SO}}_{4}$ sat. $K_{2}{\text{SO}}_{4}$), the rate of the metal dissolution (measured as the current) exhibits periodic oscillations (*35*). Applying a combination of individual, population, and global resistances yields a modular network structure of coupled electrochemical reactions. The individual resistances ($R_{\text{ind}}$) are connected directly to the electrodes and the two population resistances ($R_{p}$) are connected to the individual resistances for electrodes 1 to 40 and 41 to 80 for populations 1 and 2, respectively. Moreover, a global resistance ($R_{c}$) is connected to the two population resistances. Further details on the experiment are available in the Materials and Methods.

The resistors induce a strong coupling within the two populations (*K*) and a weak coupling between them (ε*K*). In particular, $R_{p}$ induces a coupling $K_{p}=R_{p}/\left[R_{\text{ind}}\left(R_{\text{ind}}+40R_{p}\right)\right]$ within each population. In addition, $R_{c}$ induces a global (all-to-all) coupling $K_{c}=R_{c}/\left[\left(R_{\text{ind}}+40R_{p}\right)R_{\text{eq}}\right]$, where $R_{\text{eq}}=R_{\text{ind}}+40R_{p}+80R_{c}$. Hence, the total intra- and interpopulation coupling can be calculated as $K=K_{p}+K_{c}$ and $\mathrm{\varepsilon K}=K_{c}$, respectively.

Figure 4B shows the synchronization pattern, including phase differences, for a typical configuration ($R_{\text{ind}}=580$ ohm, $R_{p}=9$ ohm, and $R_{c}=0.5$ ohm) with intrapopulation coupling *K* = 17.0 μS and cross-coupling factor ε = 0.03. In population 1, the oscillators are in-phase synchronized (forming a one-cluster state) with a frequency of 0.378 Hz. In population 2, the oscillators form a two-cluster state in which the clusters are antiphase synchronized with a common frequency of 0.406 Hz. Because the one- and the two-cluster populations have a frequency difference of 28 mHz, the phase difference between the populations drifts approximately linearly. This behavior confirms the existence of a weak chimera state (*37*, *38*).

To explore the impact of frequency detuning on the synchronization pattern, we note that the intrinsic frequencies of the oscillators can be varied by changing the individual resistances ($R_{\text{ind}}$). As these resistances are increased, the intrinsic frequencies of the oscillators increase at the approximately linear rate of 0.35 mHz/ohm (see Materials and Methods). Figure 4C shows the synchronization pattern when $R_{\text{ind}}$ of each wire in population 1 is increased by 170 ohm relative to $R_{\text{ind}}$ in population 2 (i.e., ΔR=170 ohm). While populations 1 and 2 retain their one- and two-cluster states, respectively, they now evolve at the same frequency of 0.409 Hz. Therefore, the experiments confirm our prediction that detuning the intrinsic frequencies between the two populations can result in globally frequency-synchronized dynamics.

Figure 4D shows the frequencies of the individual oscillators (relative to the first oscillator in population 1) for a range of values of ΔR. Without detuning (i.e., ΔR=0 ohm), there is a 28 mHz frequency difference between the oscillators in populations 1 and 2. When the intrinsic frequency in population 1 is increased by increasing ΔR, the frequency difference decreases; for ΔR=100 ohm, the frequency difference is 7 mHz. When ΔR is further increased, the system reaches a globally synchronized state (${\Delta \Omega }_{21}=0$ mHz) for the range 150ohm≤ΔR≤190ohm. For ΔR>190ohm, the frequency difference becomes negative, and the two populations are no longer synchronized with each other. This confirms that the detuning-induced synchronization takes place through an entrainment process similar to those resulting from an Arnold tongue in Fig. 2D.

**Fig. 5.**
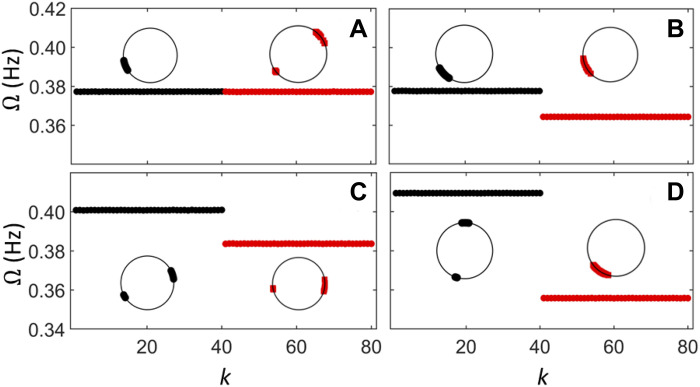
Interplay between frequency detuning and initial conditions in electrochemical experiments. Each panel shows the frequency indexed by the oscillator number in population 1 (black, 1 to 40) and 2 (red, 41 to 80) for the parameters used in Fig. 4C. The panels differ by their initial conditions, which lead to stable states with phases in population σ forming either one (I) or two (II) clusters. (**A**) Global frequency-synchronized state with a I-II phase configuration. (**B** to **D**) Desynchronized states with a I-I, II-II, and II-I configuration, respectively. The insets show phase snapshots in each population.

Thus far, all presented experiments had initial conditions in which populations 1 and 2 were close to one- and two-cluster states, respectively. Figure 5 presents experimental results for several different initial conditions at the fixed detuning ΔR=170 ohm, where each main panel shows the frequency of the individual oscillators and the inset depicts a snapshot of the final state for the given initial condition. Global frequency synchronization is observed for initial conditions close to the one-cluster state for population 1 and the two-cluster state for population 2 (Fig. 5A), as in Fig. 4C. In contrast, when both populations have similar initial conditions (one cluster in Fig. 5B and two clusters in Fig. 5C), population 1 exhibits a higher frequency and thus global synchronization is not observed. As shown in Fig. 5D, the frequency difference is even larger (53 mHz) when the initial conditions in Fig. 5A are swapped between the two populations. Different initial conditions lead to qualitatively different outcomes due to multistability. The experiments reveal an interplay between frequency detuning and the initial conditions: The detuning used in Fig. 5 does not yield global frequency synchronization for identical initial phases but it does for initial (and final) split phases.

We also extracted the experimental interaction function using data corresponding to the desynchronized state shown in Fig. 5B. The resulting function, presented in fig. S11, aligns with theoretical predictions: It supports stable one- and two-cluster states with nonzero frequency differences. The experimentally obtained interaction function *g* is dominated by a second-harmonic component, with additional contributions from the first and third harmonics. These findings confirm the theoretical insight that frequency synchronization under detuning can occur even when the first harmonic is weak, provided that *g* contains a sufficiently strong, phase-attracting second-harmonic component.

## DISCUSSION

This work shows that heterogeneity-induced frequency synchronization can be observed for a general class of phase oscillator networks, enabling the synchronization of otherwise asynchronous states. This is achieved by allowing the stabilized states to be frequency synchronized without constraining them to exhibit identical phases. This effect constitutes a generalization for synchronization in multistable modular networks of the recently discovered phenomenon of converse symmetry breaking (*14*, *15*, *45*, *46*), in which dynamical order is induced by system disorder. Unlike previous work, this study shows that heterogeneity can promote global frequency synchronization of otherwise cluster-synchronized or chimera states and even when: (i) the coupled systems are one-dimensional phase oscillators, (ii) the heterogeneity is implemented directly on the intrinsic frequencies, and (iii) the same heterogeneity is implemented for all oscillators in a network module. The latter shows that even low-dimensional parameter manipulation can realize the full potential of detuning-induced synchronization. We identified three key ingredients for this phenomenon to occur: coupling involving higher harmonics, phase frustration, and weak coupling between the modules, which are all features common to many systems. Higher-order harmonics are required to describe nonlinear terms for phase reduction away from Hopf and homoclinic bifurcations (*36*, *47*), phase frustration emerges naturally in the presence of amplitude-dependent oscillation periods and coupling delays (*48*, *49*), and real networks are known to often exhibit modular structure (*50*). As such, many biological and physical oscillator networks have the potential to exhibit frequency synchronization induced by frequency detuning, with important implications for their stability, optimization, and control.

## MATERIALS AND METHODS

### Theory for more general interaction functions

To show that frequency detuning can induce frequency synchronization for a range of phase frustration (or phase shift) parameter α of the phase model, we consider a general phase interaction function with two harmonics given by

g(ϕ)=sin(ϕ+α)−rsin(2ϕ+β) (8)

Here, α and β are the phase shifts of the first and second harmonic, respectively, and *r* is the amplitude of the second harmonic relative to the first. In the main text, the special case β=2α is considered. Here, we separate contributions from α and β to better represent the role of the phase shifts and second-harmonic amplitude. For $\beta \in \left[\frac{\pi }{2},\frac{3\pi }{2}\right]$ and r>0, the attracting second harmonic can lead to coexisting stable one- and two-cluster states; explicit conditions can be stated in terms of the derivative of *g* (*40*).

The frequency difference ${\Delta \Omega }_{21}^{u}$ between the phase synchronized solution = 0 and the two-cluster solution Δψ=π is

$${\Delta \Omega }_{21}^{u}=-g\left(0\right)+g\left(\pi \right)=-2\text{sin}\left(\alpha \right)$$ (9)

independent of *r* and β. This means that the frequency difference is maximal for $\alpha =\pm \frac{\pi }{2}$ and detuning is required for frequency synchronization to emerge.

As outlined in the main text, the existence of an equilibrium $\left(\psi ,\psi \prime \right)=\left(\psi^{*},\psi^{*}-\pi \right)$ and thus of $\psi^{*}$ such that $0=\Delta \omega_{12}-{\Delta \Omega }_{21}^{u}+\varepsilon \left[g\left(\psi^{*}\right)-g\left(-\psi^{*}\right)+g\left(\psi^{*}-\pi \right)-g\left(-\psi^{*}+\pi \right)\right]$, gives a condition for frequency synchronization. For the phase interaction function (*8*), this condition reads

$$\begin{array}{c} \Delta \omega_{12}-{\Delta \Omega }_{21}^{u}\\ =\varepsilon \left[2\text{sin}\left(\psi^{*}\right)\text{cos}\left(\alpha \right)-2\text{cos}\left(\psi^{*}\right)\text{sin}\left(\alpha \right)-4r\text{sin}\left(2\psi^{*}\right)\text{cos}\left(\beta \right)\right] \end{array}$$ (10)

If the second harmonic is dominant (i.e., r≫0) and β≈−π, we can focus on the last term to obtain the condition

$$|\Delta \omega_{12}-{\Delta \Omega }_{21}^{u}|\leq 4\varepsilon r\text{cos}\left(\beta \right)$$ (11)

This coincides with Eq. 4 for β=−π. Thus, the phase locking condition depends on both the strength and phase shift of the second harmonic.

### Phase model simulations for more complex networks

The simulations in Fig. 3 and figs. S8 to S10 on more general networks are performed using the following phase model

$${\dot{\theta }}^{k}=\omega^{k}+\sum_{l=1}^{N_{\text{tot}}}K_{\mathrm{kl}}\;g\left(\theta^{l}-\theta^{k}\right)$$ (12)

where $N_{\text{tot}}$ is the total number of oscillators and $K_{\mathrm{kl}}$ is the coupling strength between oscillators *l* and *k*; as before, *g* is the interaction function, and $\theta^{k}$ and $\omega^{k}$ are, respectively, the phase and intrinsic frequency of oscillator *k*. This model reduces to Eq. 1 in the case of two populations of *N* globally coupled oscillators, given suitable choices of $K_{\mathrm{kl}}$, even though the individual populations are not separately indexed in this formulation.

To specify $K_{\mathrm{kl}}$, each oscillator is assigned to the respective population σ, and the coupling within and across populations is assumed to occur through a mean field. Specifically, the coupling strength between nodes *l* and *k* in the same population is $K_{\mathrm{kl}}=2/{d_{k}}^{\prime }$, where ${d_{k}}^{\prime }$ is the (in-)degree of node *k* within its population subgraph. Similarly, the coupling strength between nodes *l* and *k* in different populations is $K_{\mathrm{kl}}=2\varepsilon /{d_{k}}^{\prime \prime }$, where ${d_{k}}^{\prime \prime }$ is the degree of node *k* within the subgraph formed by interpopulation couplings.

The intrinsic frequencies are set to $\omega^{k}=0$ for populations in a two-cluster state and $\omega^{k}=\Delta \omega_{12}$ for populations in a one-cluster state. The detuning is thus implemented by increasing the intrinsic frequency of the oscillators in the populations with one-cluster states. All simulations are performed using ε = 0.1 and *g* as defined in Eq. 3 for *r* = 1 and α=−π/2, where the letter leads to self-coupling as in the other networks considered in this study.

### Experimental setup

The experimental setup consists of a standard three-electrode cell with a counter (platinum-coated titanium rod), reference ($\text{Hg}/{\text{Hg}}_{2}{\text{SO}}_{4}$ sat. $K_{2}{\text{SO}}_{4}$), and a working electrode array (nickel wires of 1 mm in diameter) connected to a potentiostat (AC Instruments Gill AC). The electrolyte was 3 M $H_{2}{\text{SO}}_{4}$ held at a constant temperature of 10°C. The electrode array consists of 80 nickel wires embedded in epoxy with a spacing of 3 mm, so the electrochemical reaction only takes place on the surface end exposed to the solution. We set the circuit potential at *V* = 1.260 V and measured the current with an acquisition rate of 200 Hz using a Labview National Instruments interface.

### Effect of individual resistances on intrinsic frequencies

We measured the effect of the individual resistances on the intrinsic frequencies of all 80 uncoupled oscillators in our experiment (Fig. 4A). In these measurements, only individual resistors were used (i.e., $R_{p}=R_{c}=0$ ohm). The intrinsic frequencies increased approximately linearly with a slope of 0.35 mHz/ohm as the individual resistances were increased (fig. S1).

## Supplementary Materials

### The PDF file includes:

Supplementary Text Figs. S1 to S11 Legend for movie S1 References
