# Supplementary material for: Frequency synchronization induced by frequency detuning
Source: Sci Adv. 2025 Jun 11;11(24):eadu4114. doi: 10.1126/sciadv.adu4114 (PMC13109953; doi:10.1126/sciadv.adu4114)
Supplement: Supplementary file 1 — Supplementary Text Figs. S1 to S11 Legend for movie S1 References [file sciadv.adu4114_sm.pdf]

Science Advances
AAAS

Supplementary Materials for

Frequency synchronization induced by frequency detuning

Jorge Luis Ocampo-Espindola *et al.*

Corresponding author: István Z. Kiss, istvan.kiss@slu.edu

Sci. Adv. **11**, eadu4114 (2025)
DOI: 10.1126/sciadv.adu4114

The PDF file includes:

Supplementary Text
Figs. S1 to S11
Legend for movie S1
References

Other Supplementary Material for this manuscript includes the following:

Movie S1

Supplementary Text

Simulations with the nickel electrodissolution model

We used a nickel electrodissolution model proposed by Haim *et al.* (*51*) under potentiostatic conditions (*52, 35*) to simulate detuning-induced synchronization in Fig. 1B. The behavior of two populations of 2 oscillators was simulated with a strong electric coupling within the populations (K) and a weak coupling (εK) between the populations. Each oscillator is described by two variables, the electrode potential e and the total surface coverage of nickel oxide and hydroxide θ. The two variables, e and θ, are governed by the following equations:

$$\frac{de_l}{dt} = \frac{V - e_l}{R_1} - J_F(e_l, \theta_l) + K \sum_{j=1}^{2} (e_j - e_l) + \varepsilon K \sum_{j=3}^{4} (e_j - e_l) \quad \text{for} \quad l = 1, 2, \tag{S1}$$

$$\frac{de_l}{dt} = \frac{V - e_l}{R_2} - J_F(e_l, \theta_l) + \varepsilon K \sum_{j=1}^{2} (e_j - e_l) + K \sum_{j=3}^{4} (e_j - e_l) \quad \text{for} \quad l = 3, 4, \tag{S2}$$

$$\Gamma \frac{d\theta_l}{dt} = \frac{\exp(0.5 e_l)}{1 + C_h \exp(e_l)} (1 - \theta_l) - \frac{b\, C_h \exp(2 e_l) \theta_l}{c\, C_h + \exp(e_l)} \quad \text{for} \quad l = 1, \ldots 4, \tag{S3}$$

where oscillators $l = (1, 2)$ and $(3, 4)$ belong to populations 1 and 2, respectively, $V = 25.18$ is the dimensionless circuit potential, R_1 and R_2 are the dimensionless (equivalent) resistances of populations 1 and 2, respectively, and t is the dimensionless time. Here, $\Gamma = 0.01$ is the surface capacity, $J_F(e, \theta)$ is the Faraday current density

$$J_F(e, \theta) = \left[\frac{C_h \exp(0.5 e)}{1 + C_h \exp(e)} + a \exp(e) \right] (1 - \theta), \tag{S4}$$

and $C_h = 1600$, $a = 0.3$, $b = 6 \times 10^{-5}$, and $c = 1 \times 10^{-3}$ are kinetic parameters.

With the resistances for the two populations set to $R_1 = R_2 = 20.00$ (i.e., without detuning), the system exhibits a weak chimera state. In this state, one population remains in-phase while the oscillators in the other population are in anti-phase, and there is a nonzero frequency difference between the two populations (Fig. S2A). Fig. S2B shows the behavior with frequency detuning, in this case for $R_1 = 20.23$ while keeping $R_2 = 20.00$, the system exhibits global frequency synchronization in which the oscillators in population 1 are in-phase synchronized and those in population 2 are anti-phase synchronized (Fig. S2B).

Fig. 1B shows the frequencies of the oscillators by changing the individual resistance R_1 from 20.00 to 20.29 in small steps. The amount of heterogeneity was defined by the resistance change

$\Delta R = R_1 - R_2$ (the sign of the heterogeneity change was chosen to be positive for the conditions considered in Fig. 1B). For $\Delta R = 0$, there is a chimera state with a positive frequency difference between the populations (18.6×10^{-4}). When ΔR is increased, the frequency difference decreases. Frequency synchronization was achieved for the region $0.22 \leq \Delta R \leq 0.24$. Further increasing ΔR causes the two populations to desynchronize relative to each other.

As shown in Fig. S3A, Fig. 1B concerns initial conditions in which populations 1 and 2 are close to one- and two-cluster states, respectively. The impact of the same frequency detuning for several different initial conditions is shown in Fig. S3B-D. This is consistent with the experimental results in Fig. 5.

Additional simulations were performed to numerically estimate the phase interaction function g in Eq. (1) that corresponds to the dynamics of system (S1)–(S3) with weak coupling. For this purpose, the simulations were performed with initial conditions close to the I-I configuration state (one cluster in each population) for $N = 2$, $\Delta R = 0.03$, and the other parameters as specified earlier. The interaction function is obtained by representing the dynamics of each population by the average over the corresponding e_l and plotting the instantaneous frequency of population σ (corrected by the natural frequency) $\dot{\theta}_\sigma - \omega_\sigma$ versus $\Delta\theta$. The resulting interaction function is shown in Fig. S7A–B along with the magnitude of its numerically determined Fourier harmonics up to 4th order.

Simulations with the integrate-and-fire model

We used an autocatalytic integrate-and-fire model (*36*) to simulate detuning-induced synchronization in Fig. 1C. The model consists of a population of four oscillators with a state variable u_l, for $l = 1, 2, 3, 4$, and a parameter p_l for each oscillator that determines whether the variable is increasing or decreasing. The oscillators are governed by the integrate-and-fire equations

$$\tau_l \frac{du_l}{dt} = p_l u_l - (1 - p_l) u_l B + p_l K(\mu_l - u_l), \tag{S5}$$

where $l = 1, 2$ and $3, 4$ correspond to populations 1 and 2, respectively, and K is the coupling parameter. When the variable u_l reaches 1 from below, p_l is set to 0, and the variable starts to decrease; when the variable u_l reaches A from above, p_l is set to 1, and the variable starts to increase. The parameters A, B, and τ describe oscillator properties, where τ_l defines the timescale of oscillator l.

The variables are coupled to the corresponding mean fields as $\mu_1 = \mu_2 = \frac{u_1+u_2+\gamma(u_3+u_4)}{2+2\gamma}$ and $\mu_3 = \mu_4 = \frac{u_3+u_4+\gamma(u_1+u_2)}{2+2\gamma}$, where γ is the cross-coupling factor between the populations. Note that the oscillators have a refractory period in the sense that there is no coupling when u_l is decreasing. That is, the coupling is present only in the excitatory period (i.e., when u_l is increasing). This model has proved useful in describing the dynamics of oscillators close to a saddle-loop (i.e., homoclinic) bifurcation (*36*).

Heterogeneities in parameter τ_l emulate frequency detuning in the system by setting different timescales (intrinsic periods) for the oscillators. The timescales are assumed to be the same within each population, i.e., $\tau_1 = \tau_2$ and $\tau_3 = \tau_4$. The heterogeneities are quantified through $\Delta\tau = \tau_3 - \tau_1$ (the sign was once again chosen to be positive for the conditions considered in Fig. 1C). Fig. S4A shows that without frequency detuning (i.e., $\Delta\tau = 0$) the system exhibits a weak chimera state. As in the case of the nickel electrodissolution model (Fig. S2), the oscillators in population 1 are in-phase synchronized and those in population 2 are anti-phase synchronized, with the two populations exhibiting different frequencies.

Fig. S4B shows the corresponding behavior with frequency detuning, in this case for $\Delta\tau = 0.016$. The system exhibits a globally synchronized state in which the oscillators in population 1 are in-phase and those in population 2 are in anti-phase, but with both populations now exhibiting the same frequency.

Fig. 1C shows the frequencies of the oscillators by changing $\Delta\tau$ in small steps from zero to 0.020. For $\Delta\tau = 0$, there is a weak chimera state with a frequency difference of 9×10^{-3} between the populations. When $\Delta\tau$ is increased, the frequency difference between populations decreases and a frequency synchronized region appears for $0.15 \leq \Delta\tau \leq 0.17$. Increasing $\Delta\tau$ beyond $\Delta\tau = 0.17$ leads to frequency desynchronization, which is similar to the behavior observed for the nickel electrodissolution model.

We also numerically estimated the phase interaction function for the integrated-and-fire model following the same procedure used for the nickel electrodissolution model. In this calculation, we use $N = 2$, $\Delta\tau = 0.1$, $K = 1.3$, and the previously specified values for the other parameters. The interaction function is shown in Fig. S7C–D along with the magnitude of the Fourier harmonics up to 4th order.

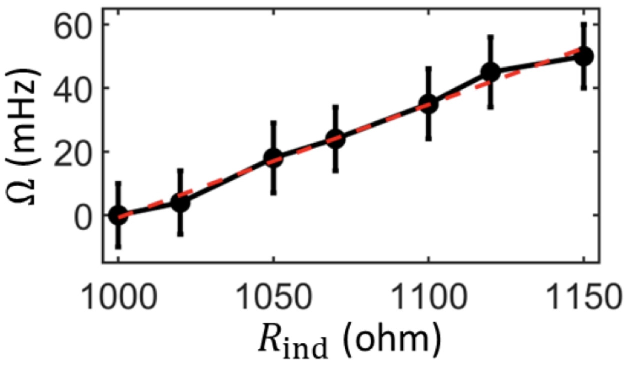

Figure S1: **The effect of individual resistances on the intrinsic frequencies of the oscillators (relative to the frequency for R_{ind} = 1000 ohm).** The dots mark the mean values and the error bars represent the standard deviations for 80 nickel electrodes, which is is approximately the same for all points. The straight dashed line indicates a linear trend.

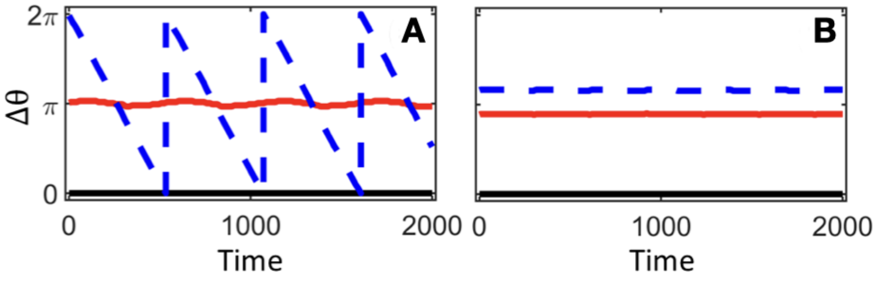

Figure S2: **Detuning-induced synchronization in the nickel electrodissolution model.** (**A**) Time series of the phase differences for a weak chimera (frequency-desynchronized) state in the absence of detuning ($\Delta R = 0$). (**B**) Phase difference for a globally synchronized state in the presence of detuning ($\Delta R = 0.23$). In both panels, the curves indicate the phase difference $\Delta\theta = \theta_\sigma^k - \theta_\kappa^j$ in population 1 (black line), in population 2 (red line), and between the two populations (blue dashed line). The other parameters are $N = 2$, $K = 0.002$, and $\varepsilon = 0.05$.

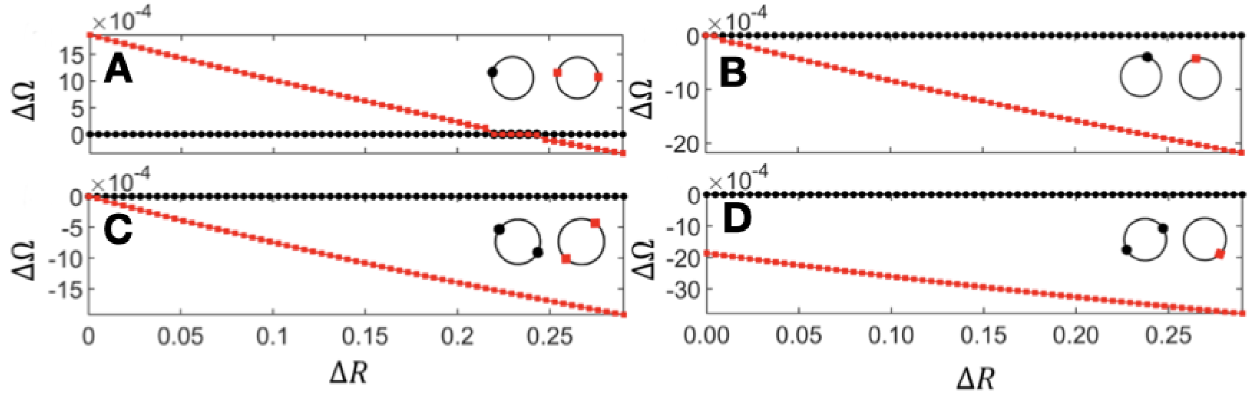

Figure S3: **Interplay between frequency detuning and initial conditions in the nickel electrodissolution model.** The oscillator frequencies in population 1 (black) and population 2 (red) (relative to oscillator 1 in population 1) are shown as functions of ΔR for different initial conditions. The insets depict phase snapshots in each population, showing the convergence to either one (I) or two (II) clusters in each population. (**A**) Detuning-induced synchronization for a I–II phase configuration (as considered in Fig. 1B). (**B**)-(**D**) Detuning-induced desynchronization for I–I, II–II, and II–I configurations, respectively. The parameters are the same as in Fig. S2.

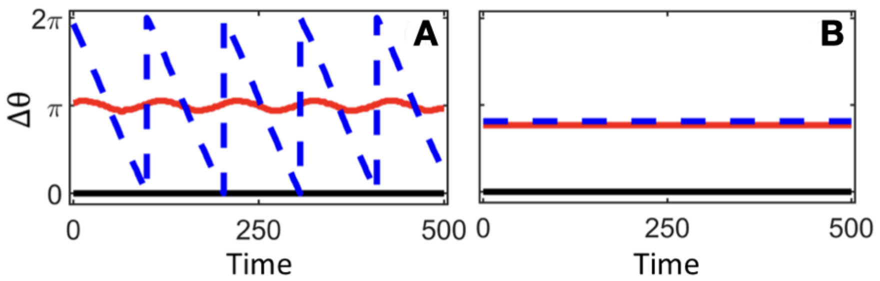

Figure S4: **Detuning-induced synchronization in the integrate-and-fire model.** (**A**) Time series of the phase differences for a weak chimera state in the absence of detuning ($\Delta\tau = 0$). (**B**) Phase difference for a globally synchronized state in the presence of detuning ($\Delta\tau = 0.016$). In both panels, the curves indicate the phase difference $\Delta\theta = \theta_\sigma^k - \theta_\kappa^j$ in population 1 (black line), in population 2 (red line), and between the two populations (blue dashed line). The other parameters are $N = 2$, $K = 0.1$, $\gamma = 0.1$, $A = 0.18$, and $B = 17.0$.

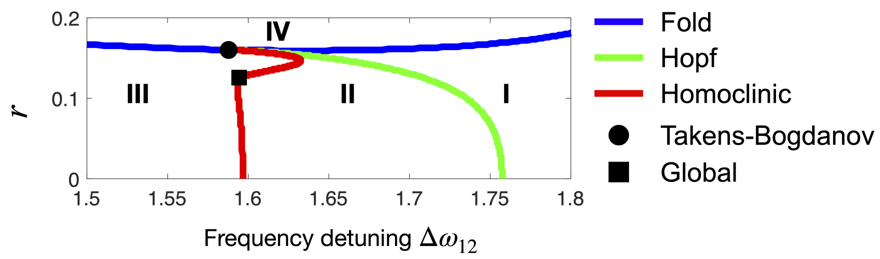

Figure S5: **Detailed bifurcation diagram in Fig. 2E.** Magnified diagram of the inset in Fig. 2E (dashed red box). The typical qualitative dynamical behavior in the various regions is as follows: frequency synchronization with constant phase differences in both populations (region I), frequency synchronization with zero phase differences in the one-cluster population and oscillatory phase differences in the two-cluster population (region II), and unsynchronized dynamics with zero phase differences in the one-cluster population and oscillatory phase differences in the two-cluster population (regions III and IV).

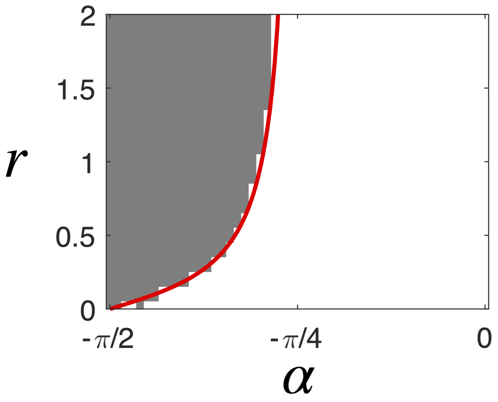

Figure S6: **Parameter region of detuning-induced frequency synchronization in the phase model.** Phase diagram for the model in Eqs. (1) and (3) with $N = 2$. Gray region: combinations of r and α resulting in $|\Delta\Omega_{21}| > 0$ for $\Delta\omega_{12} = 0$ and $|\Delta\Omega_{21}| = 0$ for suitable $|\Delta\omega_{12}| > 0$, determined from simulations for $\varepsilon > 0$. For given α and r, the simulations were performed in the range of $\Delta\omega_{12} = [-3, 3]$. Red curve: theoretically predicted stability boundary above which the one- and two-cluster states are stable, calculated for $\varepsilon = 0$ as $r > -\cos(\alpha)/[2\cos(2\alpha)]$ (see (*40*) for a derivation of the formula). The numerically observed and theoretically predicted domains for detuning-induced frequency synchronization show excellent agreement.

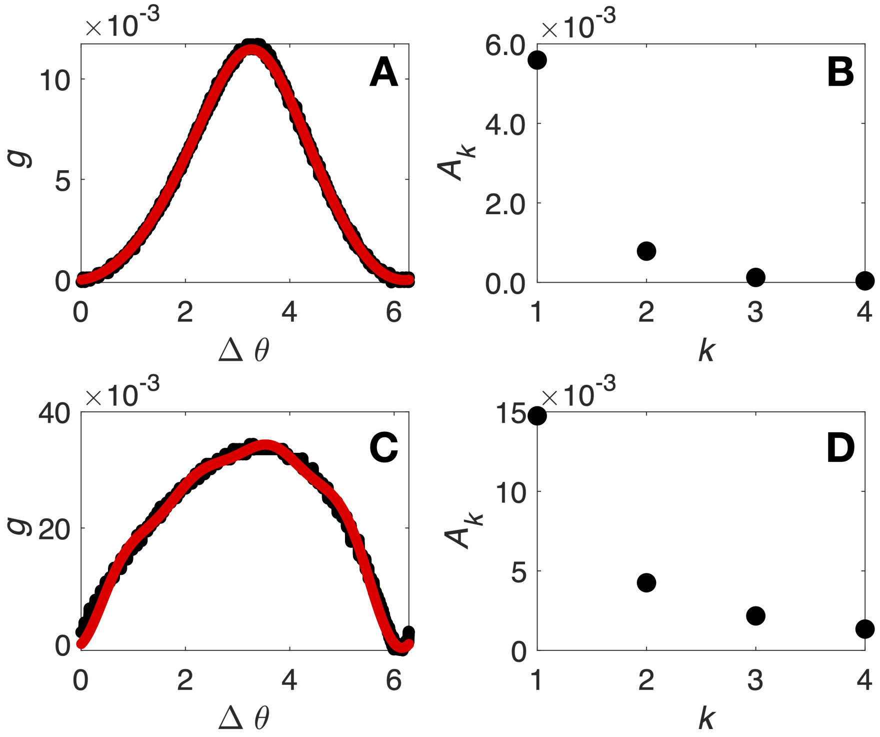

Figure S7: **Interaction functions calculated numerically for the models in Fig. 1.** Measured (black circles) and fitted (red curves) interaction functions (left) and the corresponding magnitudes of the first four Fourier harmonics (right). (**A**-**B**) Interaction function of the chemical oscillator model. The calculated g can be used to determine the stability of the one- and two-cluster states and their frequency difference [$\Delta\Omega^{\mathrm{u}}_{21} = g(\pi) - g(0)$]. The obtained interaction function, which has a dominant first harmonic and small higher harmonics, predicts a stable one-cluster state (i.e., the relevant eigenvalue is negative, $\lambda_{\max} = -2.9 \times 10^{-4}$) and a stable two-cluster state ($\lambda_{\max} = -0.8 \times 10^{-3}$) with a frequency difference of $\Delta\Omega^{\mathrm{u}}_{21} = 11 \times 10^{-3}$ between the two populations. (**C**-**D**) Interaction function of the integrate-and-fire model. Once again, the interaction function has a dominant first harmonic and predicts stable one-cluster ($\lambda_{\max} = -9.2 \times 10^{-3}$) and two-cluster ($\lambda_{\max} = -4.5 \times 10^{-3}$) states with a frequency difference of $\Delta\Omega^{\mathrm{u}}_{21} = 33 \times 10^{-3}$.

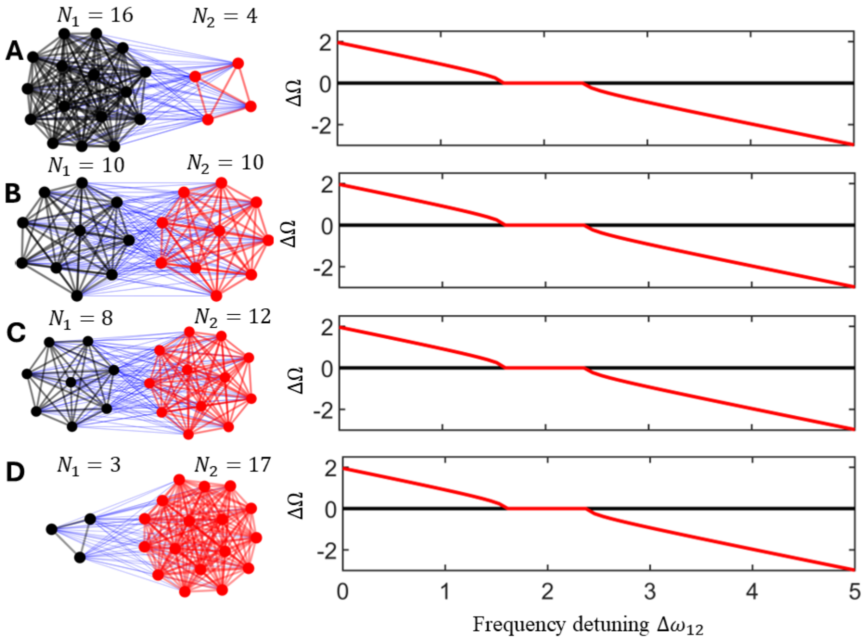

Figure S8: **Numerical simulations with phase model for populations of different sizes.** Networks with two all-to-all coupled populations of varying sizes (left) exhibit frequency synchronization over a similar range of frequency detunings (right). The simulations are for two-population modular networks with a population of size N_1 in a one-cluster state (black) and a population of size N_2 in a two-cluster state (red) for $N_1 + N_2 = 20$: (**A**) $N_1 = 16$, $N_2 = 4$; (**B**) $N_1 = 10$, $N_2 = 10$ (reference case); (**C**) $N_1 = 8$, $N_2 = 12$; and (**D**) $N_1 = 3$, $N_2 = 17$. Frequencies are shown relative to the first oscillator in the one-cluster population. For model description and other parameters, see *Materials and Methods*.

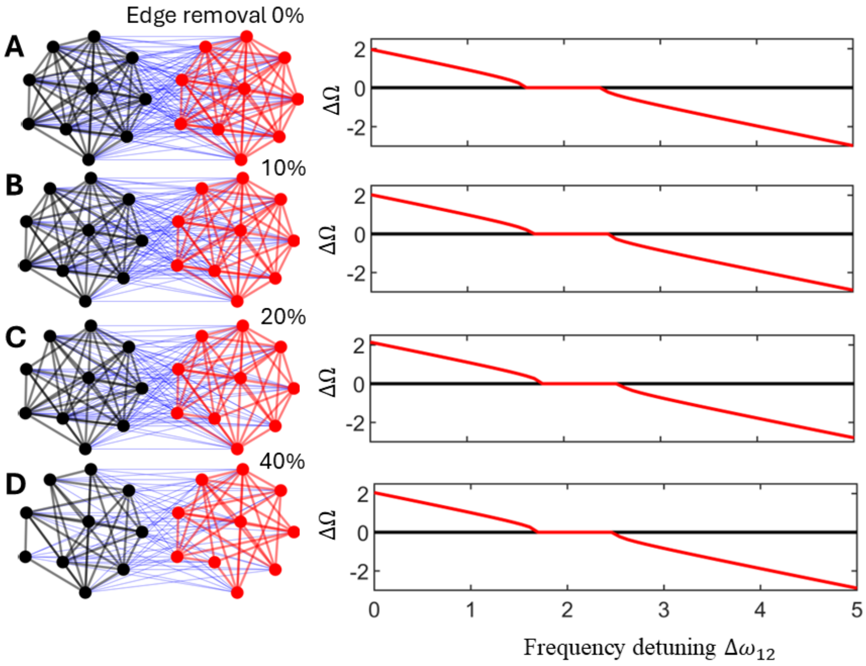

Figure S9: **Numerical simulations with phase model for random edge removal.** Networks with different extents of random edge removal (left) exhibit detuning-induced frequency synchronization over approximately the same range of frequency detunings (right). The simulations are for two populations of $N = 10$ nodes, arranged in all-to-all modular networks: (**A**) without edge removal (reference case), (**B**) with 10% of edge removal, (**C**) with 20% of edge removal, and (**D**) with 40% of edge removal. As before, we use black for the one-cluster state, red for the two-cluster state, and frequencies relative to the first oscillator of the one-cluster population.) For model description and other parameters, see *Materials and Methods*.

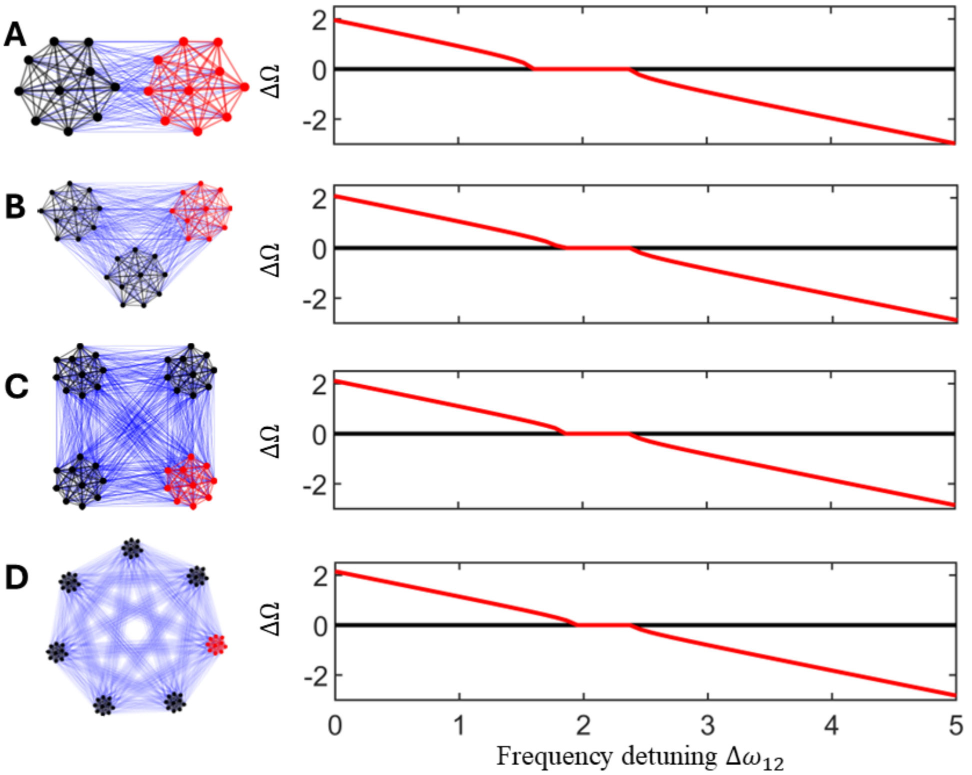

Figure S10: **Numerical simulations with phase model for a varying number of populations.** Networks with different numbers of all-to-all coupled populations (left) exhibit detuning-induced frequency synchronization (right). The simulations are for modular networks with (**A**) $N_{\text{pop}} = 2$ populations (reference case), (**B**) $N_{\text{pop}} = 3$, (**C**) $N_{\text{pop}} = 4$, and (**D**) $N_{\text{pop}} = 7$, for $N = 10$ nodes per population in all cases. We use black for one- and red for two-cluster states, and frequencies are shown relative to the first oscillator in the first one-cluster population. For model description and other parameters, see *Materials and Methods*.

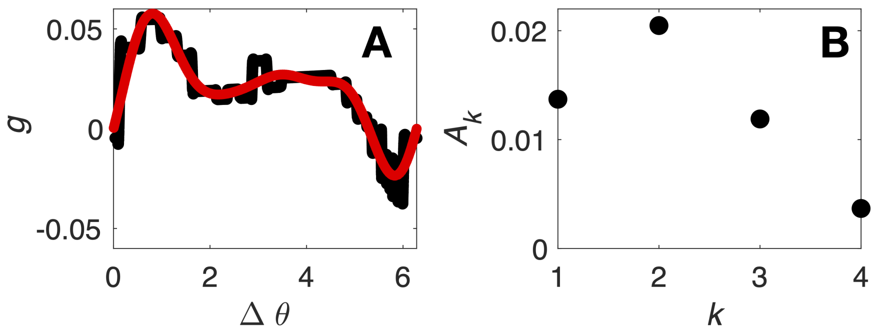

Figure S11: **Interaction function extracted numerically from the experiments.** The interaction function is estimated using data from the desynchronized state with the I-I configuration in Fig. 5B. We plot the instantaneous frequency of population σ (relative to the natural frequency) $\dot{\theta}_\sigma - \omega_\sigma$ versus $\Delta\theta$, where each population is represented by a single variable defined as the average over the currents in the population. (**A**) Measured interaction function (black circles) and fitting to a Fourier expansion to fourth order (red curve). (**B**) Magnitude of Fourier harmonics up to 4th order of the fitted interaction function in panel A. The resulting interaction function has a dominant second harmonic, with smaller magnitude first, third, and fourth harmonics. This function predicts stable one-cluster ($\lambda_{\max} = -9.4 \times 10^{-2}$) and two-cluster ($\lambda_{\max} = -1.0 \times 10^{-2}$) states with a frequency difference of $\Delta\Omega_{21}^{\mathrm{u}} = 2.5 \times 10^{-2}$ rad/s.

Caption for Movie S1. Experimentally measured dynamics of coupled electrochemical oscillators in the absence and presence of frequency detuning.

REFERENCES AND NOTES

1. A. Pikovsky, M. Rosenblum, J. Kurths, *Synchronization: A Universal Concept in Nonlinear Sciences*. (Cambridge Univ. Press) 2003.

2. S. H. Strogatz, *Sync: The Emerging Science of Spontaneous Order* (Penguin) (2004).

3. A. Arenas, A. Díaz-Guilera, J. Kurths, Y. Moreno, C. Zhou, Synchronization in complex networks. *Phys. Rep.* **469**, 93–153 (2008).

4. M. J. Panaggio, D. M. Abrams, Chimera states: Coexistence of coherence and incoherence in networks of coupled oscillators. *Nonlinearity* **28**, R67–R87 (2015).

5. O. E. Omel'chenko, The mathematics behind chimera states. *Nonlinearity* **31**, R121 (2018).

6. D. A. Wiley, S. H. Strogatz, M. Girvan, The size of the sync basin. *Chaos* **16**, 015103 (2006).

7. E. A. Martens, M. J. Panaggio, D. M. Abrams, Basins of attraction for chimera states. *New J. Phys.* **18**, 022002 (2016).

8. M. Sebek, I. Z. Kiss, Plasticity facilitates pattern selection of networks of chemical oscillations. *Chaos* **29**, 083117 (2019).

9. D. M. Abrams, L. M. Pecora, A. E. Motter, Introduction to focus issue: Patterns of network synchronization. *Chaos* **26**, 094601 (2016).

10. Z. G. Nicolaou, D. Eroğlu, A. E. Motter, Multifaceted dynamics of Janus oscillator networks. *Phys. Rev. X* **9**, 011017 (2019).

11. M. H. Matheny, J. Emenheiser, W. Fon, A. Chapman, A. Salova, M. Rohden, J. Li, M. Hudoba de Badyn, M. Pósfai, L. Duenas-Osorio, M. Mesbahi, J. P. Crutchfield, M. C. Cross, R. M. D'Souza, M. L. Roukes, Exotic states in a simple network of nanoelectromechanical oscillators. *Science* **363**, eaav7932 (2019).

12. Y. Kuramoto, *Chemical Oscillations, Waves, and Turbulence*, vol. 19 of *Springer Series in Synergetics* (Springer) (1984).

13. P. Ashwin, O. Burylko, Y. L. Maistrenko, O. V. Popovych, Extreme sensitivity to detuning for globally coupled phase oscillators. *Phys. Rev. Lett.* **96**, 054102 (2006).

14. T. Nishikawa, A. E. Motter, Symmetric states requiring system asymmetry. *Phys. Rev. Lett.* **117**, 114101 (2016).

15. Y. Zhang, J. L. Ocampo-Espindola, I. Z. Kiss, A. E. Motter, Random heterogeneity outperforms design in network synchronization. *Proc. Natl. Acad. Sci. U.S.A.* **118**, e2024299118 (2021).

16. M. Barahona, L. M. Pecora, Synchronization in small-world systems. *Phys. Rev. Lett.* **89**, 054101 (2002).

17. W. Yu, G. Chen, J. Lü, On pinning synchronization of complex dynamical networks. *Automatica* **45**, 429–435 (2009).

18. A. E. Motter, C. Zhou, J. Kurths, Network synchronization, diffusion, and the paradox of heterogeneity. *Phys. Rev. E* **71**, 016116 (2005).

19. A. M. Perego, Synchronization and amplification enabled by diversity in nonlinear optical systems and the analogy with converse symmetry breaking for coupled oscillators. *Phys. Rev. A* **106**, L031505 (2022).

20. J. F. Yang, T. A. Berrueta, A. M. Brooks, A. T. Liu, G. Zhang, D. Gonzalez-Medrano, S. Yang, V. B. Koman, P. Chvykov, L. N. LeMar, M. Z. Miskin, T. D. Murphey, M. S. Strano, Emergent microrobotic oscillators via asymmetry-induced order. *Nat. Commun.* **13**, 5734 (2022).

21. E. S. Medeiros, U. Feudel, A. Zakharova, Asymmetry-induced order in multilayer networks. *Phys. Rev. E* **104**, 024302 (2021).

22. M. I. Bolotov, V. O. Munyayev, L. A. Smirnov, G. V. Osipov, I. Belykh, Breathing and switching cyclops states in Kuramoto networks with higher-mode coupling. *Phys. Rev. E* **109**, 054202 (2024).

23. B. Garbin, J. Fatome, G.-L. Oppo, M. Erkintalo, S. G. Murdoch, S. Coen, Asymmetric balance in symmetry breaking. *Phys. Rev. Res.* **2**, 023244 (2020).

24. N. Nair, K. Hu, M. Berrill, K. Wiesenfeld, Y. Braiman, Using disorder to overcome disorder: A mechanism for frequency and phase synchronization of diode laser arrays. *Phys. Rev. Lett.* **127**, 173901 (2021).

25. R. Gast, S. A. Solla, A. Kennedy, Neural heterogeneity controls computations in spiking neural networks. *Proc. Natl. Acad. Sci. U.S.A.* **121**, e2311885121 (2024).

26. N. Lörch, S. E. Nigg, A. Nunnenkamp, R. P. Tiwari, C. Bruder, Quantum synchronization blockade: Energy quantization hinders synchronization of identical oscillators. *Phys. Rev. Lett.* **118**, 243602 (2017).

27. J. Acebrón, L. Bonilla, C. Pérez Vicente, F. Ritort, R. Spigler, The Kuramoto model: A simple paradigm for synchronization phenomena. *Rev. Mod. Phys.* **77**, 137–185 (2005).

28. K. Wiesenfeld, P. Colet, S. H. Strogatz, Frequency locking in Josephson arrays: Connection with the Kuramoto model. *Phys. Rev. E* **57**, 1563–1569 (1998).

29. D. Călugăru, J. F. Totz, E. A. Martens, H. Engel, First-order synchronization transition in a large population of strongly coupled relaxation oscillators. *Sci. Adv.* **6**, eabb2637 (2020).

30. D. M. Abrams, R. Mirollo, S. H. Strogatz, D. A. Wiley, Solvable model for chimera states of coupled oscillators. *Phys. Rev. Lett.* **101**, 084103 (2008).

31. M. R. Tinsley, S. Nkomo, K. Showalter, Chimera and phase-cluster states in populations of coupled chemical oscillators. *Nat. Phys.* **8**, 662–665 (2012).

32. E. A. Martens, S. Thutupalli, A. Fourrière, O. Hallatschek, Chimera states in mechanical oscillator networks. *Proc. Natl. Acad. Sci. U.S.A.* **110**, 10563–10567 (2013).

33. M. J. Panaggio, D. M. Abrams, P. Ashwin, C. R. Laing, Chimera states in networks of phase oscillators: The case of two small populations. *Phys. Rev. E* **93**, 012218 (2016).

34. C. Bick, M. Sebek, I. Z. Kiss, Robust weak chimeras in oscillator networks with delayed linear and quadratic interactions. *Phys. Rev. Lett.* **119**, 168301 (2017).

35. I. Z. Kiss, Y. Zhai, J. L. Hudson, Predicting mutual entrainment of oscillators with experiment-based phase models. *Phys. Rev. Lett.* **94**, 248301 (2005).

36. H. Kori, I. Z. Kiss, S. Jain, J. L. Hudson, Partial synchronization of relaxation oscillators with repulsive coupling in autocatalytic integrate-and-fire model and electrochemical experiments. *Chaos* **28**, 045111 (2018).

37. P. Ashwin, O. Burylko, Weak chimeras in minimal networks of coupled phase oscillators. *Chaos* **25**, 013106 (2015).

38. C. Bick, P. Ashwin, Chaotic weak chimeras and their persistence in coupled populations of phase oscillators. *Nonlinearity* **29**, 1468–1486 (2016).

39. S. H. Strogatz, From Kuramoto to Crawford: Exploring the onset of synchronization in populations of coupled oscillators. *Physica D* **143**, 1–20 (2000).

40. P. Ashwin, J. W. Swift, The dynamics of n weakly coupled identical oscillators. *J. Nonlinear Sci.* **2**, 69–108 (1992).

41. M. Golubitsky, I.Stewart, *The Symmetry Perspectives*, vol. 200 of *Progress in Mathematics* (Birkhäuser Verlag) (2002).

42. C. Bick, Isotropy of angular frequencies and weak chimeras with broken symmetry. *J. Nonlinear Sci.* **27**, 605–626 (2017).

43. R. Adler, A Study of Locking Phenomena in Oscillators. *Proc. IRE* **34**, 351–357 (1946).

44. E. J. Doedel, AUTO: A program for the automatic bifurcation analysis of autonomous systems. *Congr. Numer.* **30**, 265–384 (1981).

45. J. D. Hart, Y. Zhang, R. Roy, A. E. Motter, Topological control of synchronization patterns: Trading symmetry for stability. *Phys. Rev. Lett.* **122**, 058301 (2019).

46. F. Molnar, T. Nishikawa, A. E. Motter, Network experiment demonstrates converse symmetry breaking. *Nat. Phys.* **16**, 351–356 (2020).

47. H. Kori, Y. Kuramoto, S. Jain, I. Z. Kiss, J. L. Hudson, Clustering in globally coupled oscillators near a Hopf bifurcation: Theory and experiments. *Phys. Rev. E* **89**, 062906 (2014).

48. H. Sakaguchi, Y. Kuramoto, A soluble active rotater model showing phase transitions via mutual entertainment. *Prog. Theor. Phys.* **76**, 576–581 (1986).

49. I. Z. Kiss, C. G. Rusin, H. Kori, J. L. Hudson, Engineering complex dynamical structures: Sequential patterns and desynchronization. *Science* **316**, 1886–1889 (2007).

50. M. Girvan, M. E. Newman, Community structure in social and biological networks. *Proc. Natl. Acad. Sci. U.S.A.* **99**, 7821–7826 (2002).

51. D. Haim, O. Lev, L. M. Pismen, M. Sheintuch, Modeling periodic and chaotic dynamics in anodic nickel dissolution. *J. Phys. Chem.* **96**, 2676–2681 (1992).

52. I. Z. Kiss, W. Wang, J. L. Hudson, Experiments on arrays of globally coupled periodic electrochemical oscillators. *J. Phys. Chem.* **103**, 11433–11444 (1999).
